# Protective Effects of Oroxylin A on Retinal Ganglion Cells in Experimental Model of Anterior Ischemic Optic Neuropathy

**DOI:** 10.3390/antiox10060902

**Published:** 2021-06-03

**Authors:** Jia-Ying Chien, Shu-Fang Lin, Yu-Yau Chou, Chi-Ying F. Huang, Shun-Ping Huang

**Affiliations:** 1 Institute of Medical Sciences, Tzu Chi University, Hualien 970, Taiwan; 100712016@gms.tcu.edu.tw; 2 Institute of Clinical Medicine, National Yang Ming Chiao Tung University, Taipei 112, Taiwan; D55051@mail.cmuhch.org.tw; 3 Department of Molecular Biology and Human Genetics, Tzu Chi University, Hualien 970, Taiwan; 104712123@gms.tcu.edu.tw; 4 Institute of Biopharmaceutical Sciences, National Yang Ming Chiao Tung University, Taipei 112, Taiwan; 5 Department of Ophthalmology, Taichung Tzu Chi Hospital, Taichung 472, Taiwan

**Keywords:** ischemic optic neuropathy, Oroxylin A, retinal ganglion cell, microglia, Nrf2, oxidative stress, visual evoked potential (VEP), optical coherence tomography (OCT)

## Abstract

Nonarteritic anterior ischemic optic neuropathy (NAION) is the most common cause of acute vision loss in older people, and there is no effective therapy. The effect of the systemic or local application of steroids for NAION patients remains controversial. Oroxylin A (OA) (5,7-dihydroxy-6-methoxyflavone) is a bioactive flavonoid extracted from *Scutellariae baicalensis* Georgi. with various beneficial effects, including anti-inflammatory and neuroprotective effects. A previous study showed that OA promotes retinal ganglion cell (RGC) survival after optic nerve (ON) crush injury. The purpose of this research was to further explore the potential actions of OA in ischemic injury in an experimental anterior ischemic optic neuropathy (rAION) rat model induced by photothrombosis. Our results show that OA efficiently attenuated ischemic injury in rats by reducing optic disc edema, the apoptotic death of retinal ganglion cells, and the infiltration of inflammatory cells. Moreover, OA significantly ameliorated the pathologic changes of demyelination, modulated microglial polarization, and preserved visual function after rAION induction. OA activated nuclear factor E2 related factor (Nrf2) signaling and its downstream antioxidant enzymes NAD(P)H:quinone oxidoreductase (NQO-1) and heme oxygenase 1 (HO-1) in the retina. We demonstrated that OA activates Nrf2 signaling, protecting retinal ganglion cells from ischemic injury, in the rAION model and could potentially be used as a therapeutic approach in ischemic optic neuropathy.

## 1. Introduction

Nonarteritic anterior ischemic optic neuropathy (NAION) is one of the most common acute optic neuropathies in the elderly population [1], and it leads to a severe loss of visual function. The pathogenesis of NAION is probably multifactorial but remains uncertain. Predisposing risk factors such as age, small cup/disc ratio, diabetes mellitus, nocturnal hypotension, hypercholesterolemia, hypertension, coronary artery disease, and obstructive sleep apnea have been linked to NAION [2,3,4,5,6,7,8]. There is currently no safe and effective treatment for the disease. Accumulating evidence suggests that NAION results from impaired vascular autoregulation at the optic nerve head and leads to impaired ocular homeostasis [9,10,11]. These factors contribute to axonal edema and compartment syndrome for the optic nerve disc, further accelerating hypoxia, increasing oxidative stress, and inducing inflammatory processes and the consequential loss of retinal ganglion cells (RGCs) [12,13,14,15,16,17].

Neuroinflammation and microglial activation have been considered the main pathogenic mechanisms in the NAION model. Previous studies demonstrated that the invasion of extrinsic macrophages into the optic nerve (ON) triggered the release of proinflammatory cytokines or chemokines and increased intrinsic microglial activation, leading to RGC dysfunction and death in a rodent model of NAION [18,19,20]. Over the decades, inflammation initiated by NAION has been an important target for investigating the pathogenesis and developing potential treatments. Our previous study showed that Oroxylin A (OA), a flavonoid abundant in the extract of *Scutellariae baicalensis* Georgi., exerts anti-apoptotic and anti-inflammatory effects in an optic nerve crush model [21]. OA has been reported to suppress inflammatory responses [22,23], ameliorate oxidative stress damage [24,25], decrease cell apoptosis [26], inhibit thrombotic activities [27], and improve neurofunctions [28,29,30,31]. However, whether OA is effective in attenuating the neuroinflammation and microglial polarization induced by experimental anterior ischemic optic neuropathy remains to be elucidated. In this research, we evaluated the effects of OA on RGC survival, the integrity of visual function, neuroinflammation, and microglial polarization in an experimental anterior ischemic optic neuropathy model, and the potential underlying molecular mechanisms were investigated.

## 2. Materials and Methods

### 2.1. Animals and Study Design

**Figure 1:**
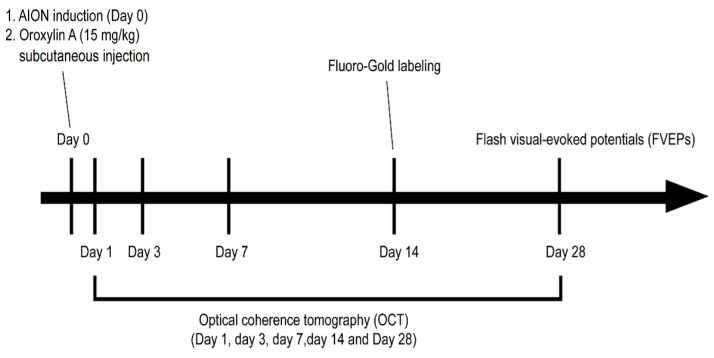
Schematic of the experimental design and procedures for Oroxylin A (OA) treatment in the rat anterior ischemic optic neuropathy (rAION) model.

Four- to six-week-old male Wistar rats (weighing 100–125 g), obtained from BioLASCO.Co. Taiwan, were used in this study. All the animal procedures were approved by the Institutional Animal Care and Use Committee (IACUC) at Tzu Chi University (No. 107008). The rats were divided into three groups: sham, AION induction treated with PBS, or AION treated with the subcutaneous injection of OA (15 mg/kg [21]; Alomone labs, Jerusalem, Israel) once immediately after laser induction. The experimental design and procedures are shown in Figure 1.

### 2.2. Anesthesia and Euthanasia

We performed all the animal experiments under general anesthesia, accomplished by an intramuscular injection of a mixture of ketamine and xylazine (100 mg/kg; 10 mg/kg body weight) [14]. The animals were kept warm on a heating pad throughout whole procedure and closely monitored until full recovery. Eye drops of 0.5% Alcaine (Alcon, Puurs, Belgium) were applied topically for local anesthesia. The pupils were dilated with Mydrin-P (Santen, Osaka, Japan) in all the animal procedures. The rats were euthanized with CO_2_ at a fill rate of 20% of the existing chamber volume per minute (5 L/min). Every effort was made to minimize distress and suffering in the animals.

### 2.3. rAION Induction

After general anesthesia, we performed AION induction via photodynamic thrombosis with an injection of 2.5 mM rose bengal (Sigma-Aldrich, St. Louis, MO, USA) (1 mL/Kg animal weight) in pH 7.4 phosphate buffered saline (PBS) [14,32]. Immediately after tail vein injection of rose bengal, the optic disc was exposed to an argon green laser (532 nm wavelength, 500 mm size, and 80 mW power) (MC-500 multicolor laser, Nidek Co., Ltd., Tokyo, Japan) at one sec/pulse for 12 sec pulses, each with a fundus contact lens. Tobradex eye ointment (Alcon, Fort Worth, TX, USA) was applied after the laser induction procedure. The rats were monitored daily for general physical health.

### 2.4. Flash Visual Evoked Potential (FVEP)

The FVEPs were measured at 28 days after rAION induction in 18 experimental rats using a visual electrodiagnostic system (Espion, Diagnosys LLC, Gaithersburg, MA, USA) as previously described [14,33]. We masked the groups in assessing the FVEP. The P1–N2 amplitude in each group was analyzed for visual function (*n* = 6 per group).

### 2.5. Retrograde Labeling of RGCs by Fluoro-Gold (FG)

The retrograde labeling procedures were described in detail in our previous reports [14]. Briefly, the RGC density of the retina was calculated at distances of 1 mm (central area) and 3 mm (mid-peripheral area) from the center of the optic disc. At least 10 randomly chosen areas (38,250 mm^2^; 225 by 170 mm^2^) in the central and mid-peripheral regions of each retina (*n* = 6 rats for each group) were counted.

### 2.6. Immunohistochemistry (IHC)

The frozen sections of ONs and retina were rinsed with PBS and then blocked with 5% normal goat serum containing 1% bovine serum albumin (BSA) for 30 min. The ON sections were labeled with anti-ED1 (CD68; 1:50; Bio-Rad, Berkeley, CA, USA), anti-2′, 3′-cyclic nucleotide 3′-phosphodiesterase (CNPase) (1:200; Abcam, Cambridge, UK), and anti-Ym1 (1:50; Abcam) primary antibodies, and the retinal sections were labeled with anti-ionized calcium binding adaptor molecule 1 (Iba1) (1:200; Abcam) and anti-interleukin 6 (IL-6) (1:1000; Abcam) primary antibodies. Those sections were then incubated with corresponding Alexa Fluor conjugated secondary antibodies. Photographs were taken using a Zeiss LSM 900 confocal system (Carl Zeiss, Oberkochen, Germany). At least six images per eye by 20× magnification were taken for quantification the ED-1 or Ym1 staining positive cell in the optic nerve and for quantification of Iba1 and IL-6 staining in the retina. To quantify the intensity of CNPase staining in optic nerve, we opened CNPase and DAPI channel in ImageJ, the intensity of CNPase and DAPI in optic nerve were measured and the CNPase generated per DAPI-positive cells were calculated.

### 2.7. Fluorescent Terminal Deoxynucleotidyl Transferase dUTP Nick end Labeling (TUNEL) Staining

Apoptotic cells in the ganglion cell layer (GCL) of the retina were detected by a TUNEL assay according to the manufacturer’s protocol (DeadEndTM Fluorometric TUNEL System; Promega Corporation, Madison, WI, USA) as described previously [4,5,6,7]. Six retina sections of each eyeball were examined using a fluorescent microscope (Zeiss), and the TUNEL-positive cells in the GCL were manually counted (*n* = 6 rats for each group).

### 2.8. In Vivo Optical Coherence Tomography (OCT) Imaging

The optic disc width and retinal nerve fiber layer (RNFL) thickness were evaluated using a Phoenix Micron IV retinal microscope with an image-guided OCT system as previously described [14,32]. We standardized the measurement by one person handled the animal and picked up the animal randomly from each group to the other person performed the OCT without knowing the animals belong to which group. The images were taken pre-rAION (Day 0) and on Days 1, 3, 7, 14, and 28 post-rAION. At least 6 clear photos were captured for each eye at different time points. The profiles of the optic disc width and RNFL thickness were analyzed using GraphPad Prism 7.0 (GraphPad Software, La Jolla, CA, USA).

### 2.9. Western Immunoblotting

The experimental procedures for immunoblotting were described in detail for previous studies [14,34]. Briefly, 30 μg of retinal extracts was separated by 10% SDS–PAGE, transferred to a PVDF membrane, blocked with 5% nonfat milk, and then incubated with anti-Nrf2 (1:200; Santa Cruz, Dallas, TX, USA), anti-NQO-1 (1:200; Abcam), anti-HO-1 (1:200; NOVUS, Centennial, CO, USA), anti-Iba1 (1:500 Abcam), anti-transforming growth factor-β (TGF-β) (1:500; Cell Signaling Technology, Danvers, MA, USA), and anti-GAPDH (1:3000; Sigma-Aldrich, St. Louis, MO, USA) primary antibodies. The blots were washed and incubated with corresponding secondary antibodies (1:10,000; Bio-Rad). The positive protein bands on the blot were detected using ECL kits (RPN2232, GE Healthcare, Piscataway, NJ, USA) and exposed using a BioSpectrum^®^ Imaging System (UVP BioSpectrum 810, UK). The signal intensity was measured and analyzed using ImageJ 1.8.0_172(U.S. National Institutes of Health, Bethesda, Maryland, USA, https://imagej.nih.gov/ij/, accessed on 28 May 2021).

### 2.10. Statistical Analysis

All the data are presented as the mean ± standard deviation (SD). Statistical analysis was performed using the Mann–Whitney U test and the Kruskal–Wallis test for comparisons between groups via GraphPad Prism 7 (GraphPad Software, La Jolla, CA, USA). *p*-values less than 0.05 were considered to indicate statistical significance.

## 3. Results

### 3.1. OA Promoted Retinal Ganglion Cell Survival

**Figure 2:**
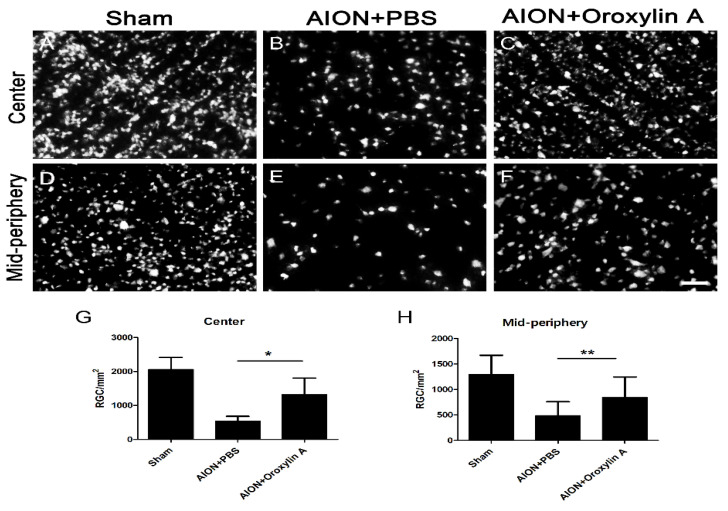
Effect of OA on the preservation of retinal ganglion cells (RGCs) after rAION. Representative images of RGC density in the retinal whole mount after rAION in each group (**A**–**F**). The RGC density of the OA-treated group was markedly higher than that of the PBS-treated group in the (**G**) central (1315 ± 490 versus 538 ± 144 cells/mm^2^, respectively) and (**H**) mid-peripheral retina (847 ± 400 versus 487 ± 274 cells/mm^2^, respectively). Results represent the means ± SDs. Scale bar, 50 μm; * *p* ≤ 0.05, ** *p* ≤ 0.01; *n* = 6.

The fluoro-gold (FG) retrograde labeling of RGCs was performed to determine the neuroprotective effect of OA on the RGCs after infarction. The mean RGC counts in the central retina in the sham, PBS-treated, and OA-treated groups were 2056 ± 361, 538 ± 144, and 1315 ± 490 cells/mm^2^, respectively (Figure 2A–C,G). The mean RGC counts in the mid-peripheral retina in the sham, PBS-treated, and OA-treated groups were 1294 ± 377, 487 ± 274, and 847 ± 400 cells/mm^2^, respectively (Figure 2D–F,H). The RGC survival rates after OA treatment were increased by 37.79% in the central retina and 26.82% in the mid-peripheral retina compared with those for the PBS-treated group. OA treatment significantly increased RGC survival after ischemic injury.

### 3.2. OA Preserved Visual Function after AION Induction

**Figure 3:**
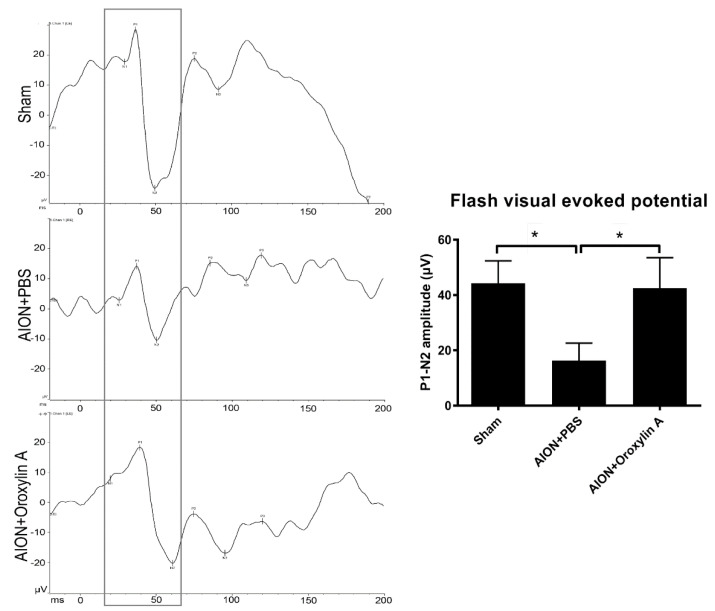
Effects of OA on visual function. Representative flash visual evoked potential (FVEP) at 4 weeks after rAION induction. The bar charts show the P1–N2 amplitudes of FVEP. The sham and OA-treated groups showed significantly higher P1–N2 amplitudes than the PBS-treated group (42.56 ± 10.91 μV versus 16.3 ± 6.32 μV, respectively). Results represent the means ± SDs. * *p* ≤ 0.05; *n* = 6.

To determine the visual function, the P1–N2 amplitudes in the flash visual evoked potentials (FVEPs) were recorded 28 days after the ischemic injury in each group. The average P1–N2 amplitudes in the sham, PBS-treated, and OA-treated groups were 44.34 ± 8.05, 16.3 ± 6.32, and 42.56 ± 10.91 μV, respectively (Figure 3). Significantly higher P1–N2 amplitude in the OA-treated group compared with that in the PBS-treated group was observed. The data indicate that the administration of OA can preserve visual function after ischemic injury.

### 3.3. OA Alleviated Optic Disc Swelling and Maintained RNFL Thickness after rAION Induction

**Figure 4:**
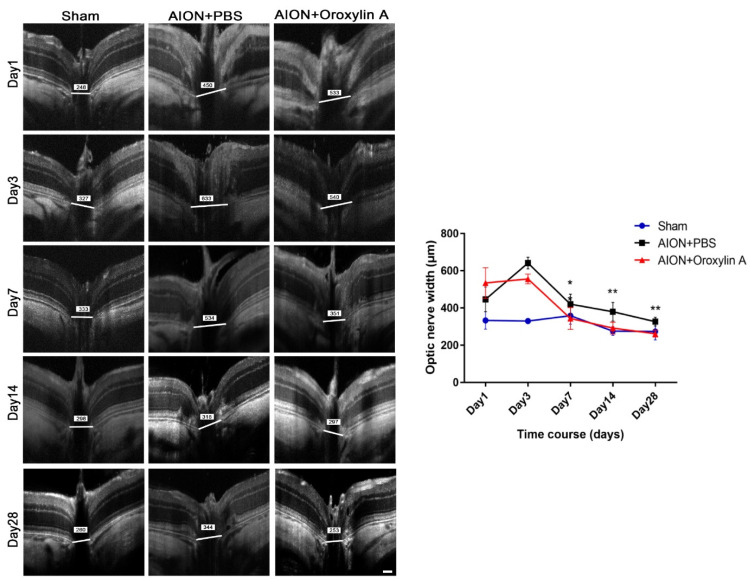
Optical coherence tomography (OCT) profiles of the optic nerve width (ONW). Representative ONW profiles for the sham, PBS-treated, and OA-treated groups on Days 1, 3, 7, 14, and 28. The ONW profile over time. The OA-treated group exhibited a significant reduction in edema on Days 7, 14, and 28 compared with the PBS-treated group. (420.73 ± 53.67 μm versus 344.11 ± 59.01 μm; 380.33 ± 49.90 μm versus 292.60 ± 31.95 μm; and 326.92 ± 23.26 μm versus 262.42 ± 17.96 μm, respectively). Results represent the means ± SDs. Scale bar, 130 μm; ** *p* < 0.01; * *p* < 0.05; *n* = 6.

**Figure 5:**
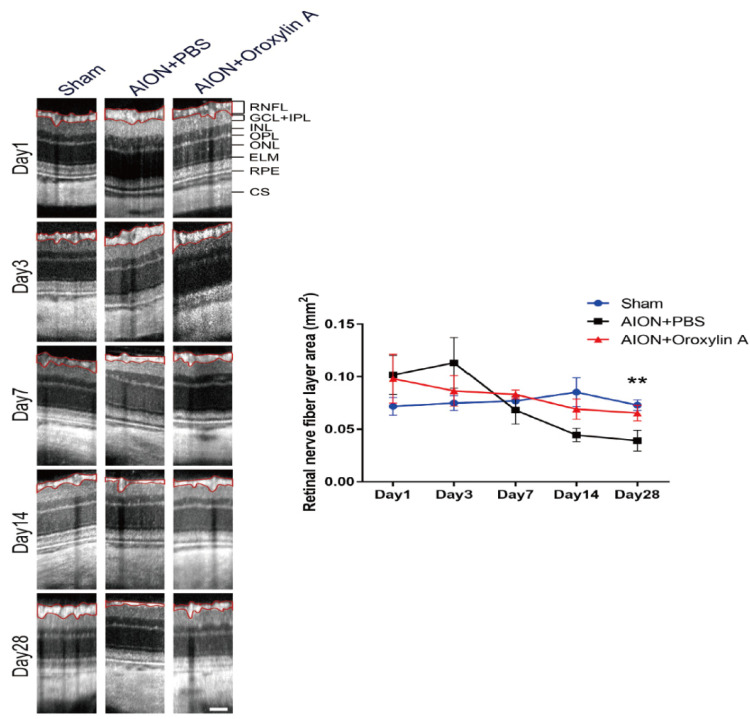
OCT profiles of the retinal nerve fiber layer (RNFL). Representative RNFL thickness measurements for the sham, PBS-treated, and OA-treated groups on Days 1, 3, 7, 14, and 28. Compared with the PBS-treated group, the OA-treated group exhibited significant preservation of the RNFL on Day 28 after infarction. (0.039 ± 0.001 mm^2^ versus 0.065 ± 0.0074 mm^2^, respectively). Results represent the means ± SDs. Scale bar, 130 μm; ** *p* < 0.01; *n* = 6. RNFL: retinal nerve fiber layer; GCL + IPL: ganglion cell layer + inner plexiform layer; INL: inner nuclear layer; OPL: outer plexiform layer; ONL: outer nuclear layer; ELM: external limiting membrane; RPE: retinal pigment epithelium; CS: choroidal stroma.

The optic nerve width (ONW) profiles of the sham, PBS-treated, and OA-treated groups were recorded on Days 1, 3, 7, 14, and 28 (Figure 4). At the acute stage of rAION, macrophage infiltration causes immediate ON edema after ischemic insult; severe edema was observed on Day 1 and had been resolved on Day 7. The spectral-domain OCT was used to quantitatively monitor changes in the ONW over time. There was a significant alleviation in ON edema in the OA-treated group compared with the PBS-treated group on Days 7, 14, and 28. The OCT profiles of the RNFL thickness measurements for the sham, PBS-treated, and OA-treated groups were determined on Days 1, 3, 7, 14, and 28 (Figure 5). Compared with the PBS-treated group, the OA-treated group exhibited significant preservation of the RNFL on Day 28.

### 3.4. OA Decreased RGC Apoptosis Induced by rAION

**Figure 6:**
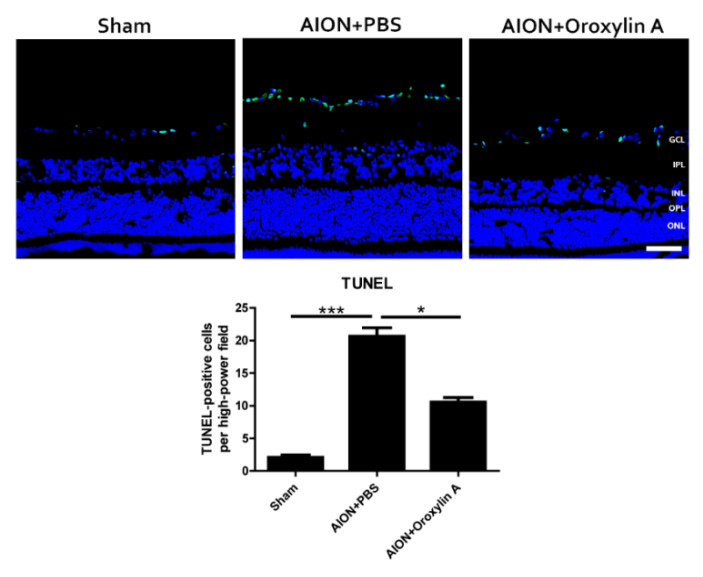
TUNEL assay in the retinal sections after rAION in each group. After ischemic injury, rats treated with OA showed significantly less TUNEL+ cells (green) than rats treated with PBS (10.6 ± 2.4 versus 20.7 ± 3.9 cells/HPF, respectively). Results represent the means ± SDs. Scale bar, 50 μm; * *p* ≤ 0.05, *** *p* ≤ 0.001; *n* = 6. GCL: ganglion cell layer; IPL: inner plexiform layer; INL: inner nuclear layer; OPL: outer plexiform layer; ONL: outer nuclear layer.

In situ TUNEL assay on retinal cross sections was performed to evaluate whether OA can protect RGCs from apoptosis (Figure 6). The numbers of TUNEL+ cells in the sham, PBS-treated, and OA-treated groups were 2.1 ± 1.1, 20.7 ± 3.9, and 10.6 ± 2.4 cells/high power filed (HPF), respectively. The OA-treated group showed significantly less TUNEL+ cells than the PBS-treated group in the retina.

### 3.5. OA Decreased Inflammatory Markers

**Figure 7:**
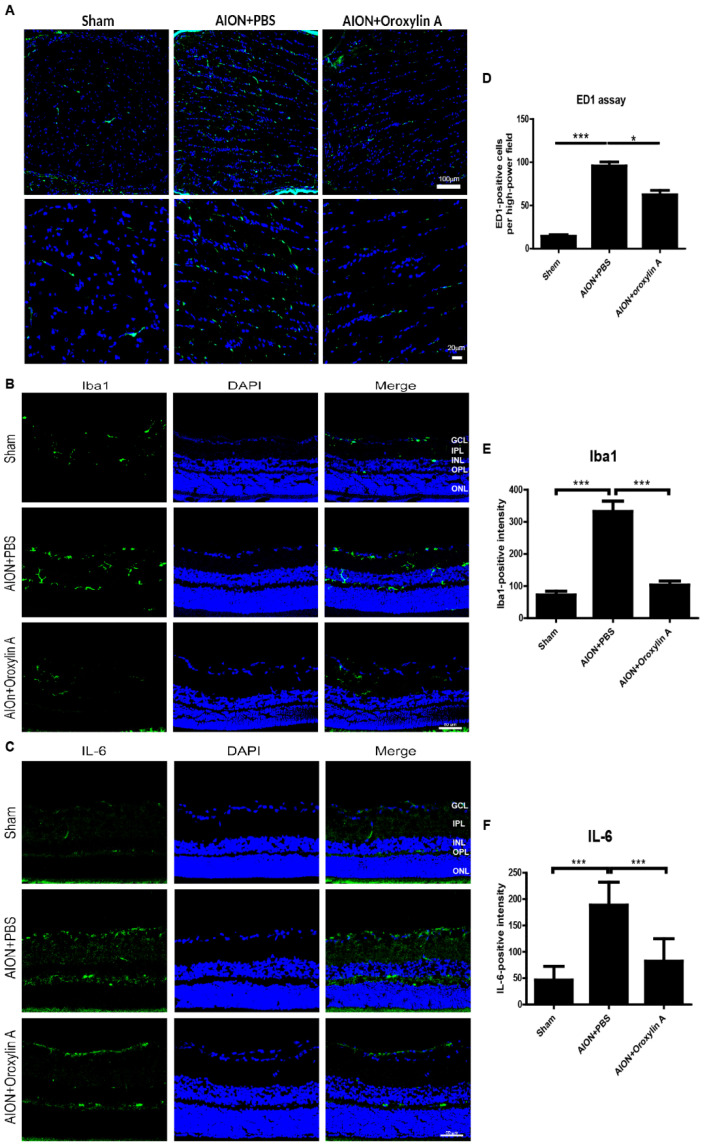
Immunohistochemistry (IHC) of ED1, Iba1, and IL-6 in optic nerves (ONs) 28 days after rAION. (**A**) the lower column indicates the ED1-positive cells (green) per HPF in the sham, PBS-treated, and OA-treated groups. (**D**) the OA-treated group showed significantly fewer ED1-positive cells in the ONs than did the PBS-treated group. The columns indicate that the numbers of ED1-positive cells (green) per HPF in the sham group, PBS-treated group, and OA-treated group were 14.56 ± 5.18, 95.75 ± 15.78, and 62.6 ± 15.39 cells/HPF, respectively. (**B**) immunohistochemistry of Iba1 in the retinas 4 weeks after rAION. (**E**) the columns indicate that the numbers of Iba1-positive cells (green) per HPF in the sham group, PBS-treated group, and OA-treated group were 72.3 ± 34.7, 332.4 ± 90.1, and 103.2 ± 38.3 cells/HPF, respectively. (**C**) immunohistochemistry of IL-6 (green) in the retinas 4 weeks after rAION. (**F**) the columns indicate that the numbers of IL-6-positive cells per HPF in the sham group, PBS-treated group, and OA-treated group were 46.6 ± 26.1, 188.6 ± 43.4, and 82.5 ± 42.5 cells/HPF, respectively. Results represent the means ± SDs. A: Scale bar, 100 μm (upper), 20 μm (lower); B and C: Scale bar, 50 μm; *n* = 6 in each group. * *p* < 0.05, *** *p* ≤ 0.001. GCL: ganglion cell layer; IPL: inner plexiform layer; INL: inner nuclear layer; OPL: outer plexiform layer; ONL: outer nuclear layer.

Blood-borne macrophages (indicated by ED1, a marker of CD38, specific for extrinsic macrophages) infiltrated the ON tissue and induced tissue inflammation after rAION induction. Immunostaining for ED1 in ON tissue was performed to evaluate whether OA treatment will attenuate the extrinsic macrophage infiltration in ONs 28 days after rAION (Figure 7A,D). The numbers of ED1-positive cells per HPF in the sham group, PBS-treated group, and OA-treated group were 14.6 ± 5.2, 97.8 ± 15.8, and 62.6 ± 15.4, respectively. The OA-treated group showed a significant reduction in ED1-positive cells in the ON compared with the PBS-treated group. These results suggested that OA treatment can reduce extrinsic macrophage infiltration in ON after ischemic injury.

The upregulation of ionized calcium binding adapter molecule 1 (Iba1), a microglial marker, indicates microglial activation during neuroinflammation [35,36]. The inflammation response to ischemic injury, the resting microglia is activated to M1 subtypes secreting one of the proinflammatory cytokines, IL-6. It will further promote microglia polarization to M1 phenotype and release more IL-6 [37]. We also performed immunohistochemistry for Iba1 and IL-6 in the retina 4 weeks after rAION. The bar chart reveals that the numbers of Iba1-positive cells per HPF in the sham group, PBS-treated group, and OA-treated group were 72.3 ± 34.7, 332.4 ± 90.1, and 103.2 ±38.3 cells/HPF, respectively (Figure 7B,E). The numbers of IL-6-positive cells per HPF in the sham group, PBS-treated group, and OA-treated group were 46.6 ± 26.1, 188.6 ± 43.4, and 82.5 ± 42.5 cells/HPF, respectively (Figure 7C,F). Our data demonstrate that OA significantly attenuated the levels of the proinflammatory cytokine IL-6 and decreased activated microglia in the retina after ischemic injury. 

### 3.6. OA Maintained the Integrity of the Myelin Sheath

**Figure 8:**
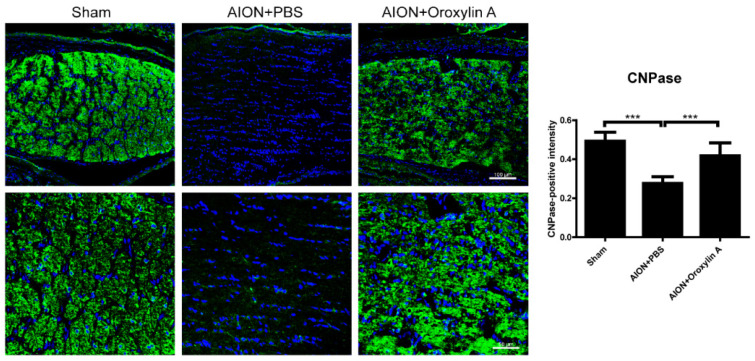
IHC of 2′, 3′-cyclic nucleotide 3′-phosphodiesterase (CNPase) in the ON 4 weeks after rAION induction. Green fluorescence represents CNPase-positive cells, and blue florescence represents nuclei. The columns indicate the intensity of the green fluorescence of CNPase-positive cells. The columns indicate that the numbers of CNPase-positive intensity (green) per DAPI in the sham group, PBS-treated group, and OA-treated group were 0.49 ± 0.047, 0.28 ± 0.035, and 0.42 ± 0.067 intensity/DAPI, respectively. Scale bar, 100 μm (upper), 50 μm (lower); Results represent the means ± SDs, *n* = 6 in each group. *** *p* ≤ 0.001.

2′,3′-Cyclic nucleotide 3′-phosphodiesterase (CNPase) is a myelination-associated enzyme and is highly expressed in the myelin-producing oligodendrocyte cells and Schwann cells [38]. Some studies have indicated that reduced CNPase levels contribute to several neurodegenerative and demyelinating diseases [39,40,41,42,43,44]. We performed IHC of CNPase in the ONs 4 weeks after infarction to evaluate the demyelinating conditions in the optic nerve (Figure 8). CNPase was highly expressed in the optic nerve in the sham group, whereas the expression was significantly reduced after rAION induction. Furthermore, OA treatment promoted CNPase expression in the ON after infarction, suggesting that OA maintained myelin integrity in the rAION model.

### 3.7. Activation of Nrf2 Signaling Contributed to the Neuroprotective Effects of OA in Retina

**Figure 9:**
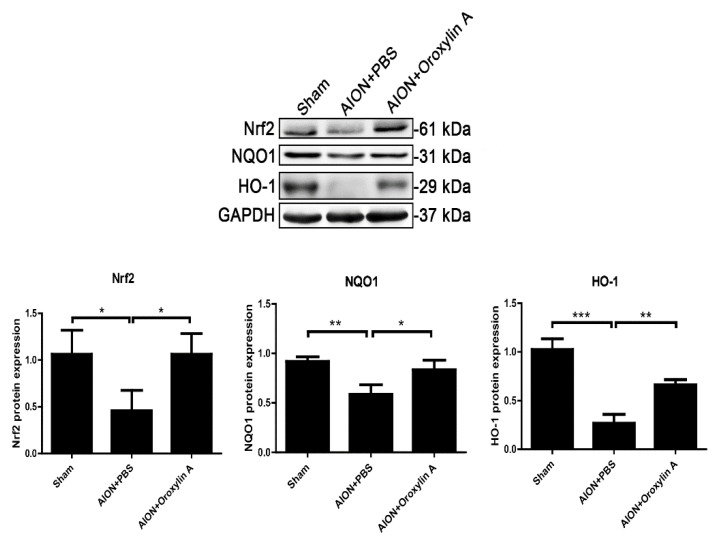
Immunoblotting analysis of Nrf2, NQO-1, and HO-1 protein expression levels 4 weeks after rAION. The bar graph shows that the expression levels of Nrf2, NQO-1, and HO-1 were decreased after rAION induction but significantly enhanced by treatment with OA. Results represent the means ± SDs and are presented as ratios with the GAPDH value for three independent experiments. * *p* ≤ 0.05, ** *p* ≤ 0.01, *** *p* ≤ 0.001.

To elucidate the molecular mechanisms involved in the neuroprotective effect of OA on RGCs, the expression of genes regulating cytoprotective responses to inflammation and oxidative stress was determined by immunoblotting analysis. We analyzed the expression of Nrf2, NQO-1, and HO-1 in the retinas after rAION. rAION induction suppressed Nrf2′s expression and that of its downstream regulated genes, NQO-1 and HO-1. OA significantly enhanced the protein expression of Nrf2, NQO-1, and HO-1 in the retinas 28 days after rAION (Figure 9).

### 3.8. OA Modulated Microglial Polarization

**Figure 10:**
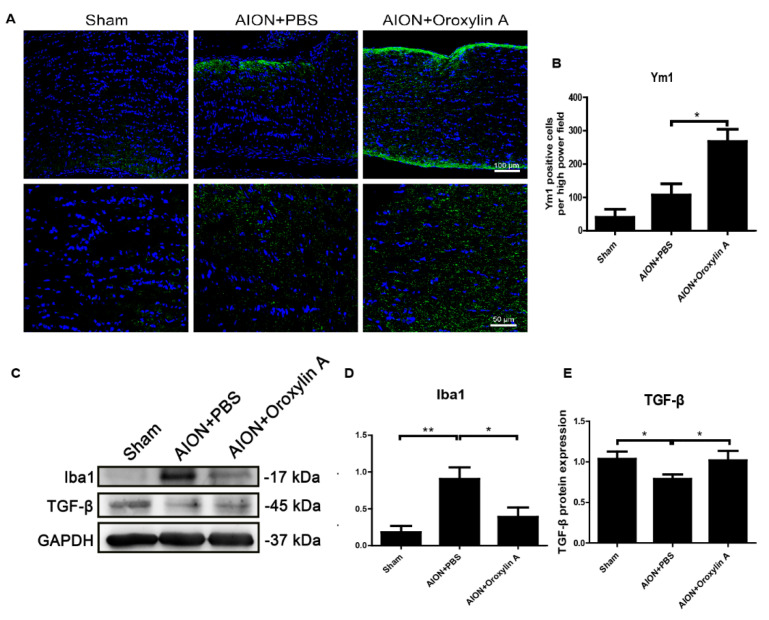
(**A**) IHC of Ym1 in the ONs 28 days after rAION. (**B**) the bar chart indicates that the numbers of Ym1-positive cells (green) per HPF in the sham, PBS-treated, and OA treated-groups were 40.3 ± 24.3, 107. 2 ± 33.5, and 268 ± 36.8, respectively. Scale bar, 50 μm, *N* = 6 in each group. (**C**) immunoblotting analysis of the expression levels of Iba1 and TGFβ in the ONs after rAION. OA suppressed Iba1 and increased TGFβ in the ONs 28 days after rAION. (**D**,**E**) quantitative analysis of (**C**). Results represent the means ± SDs and are presented as ratios with the GAPDH value for three independent experiments. Scale bar, 100 μm (upper); 50 μm (lower); * *p* ≤ 0.05, ** *p* ≤ 0.01.

Microglia and macrophages are polarized to M1 or M2 phenotypes and mediate corresponding signaling pathways in response to different pathophysiological states [45,46,47]. From Figure 6, we can see that OA suppressed extrinsic macrophage invasion and decreased intrinsic microglial activation after ischemic infarction. To further evaluate whether OA would modulate microglial polarization after ischemic optic nerve injury, we examined the protein expression levels of Ym1 and TGF-b, cytokines generated by M2 microglia, and Iba1 as a microglial marker in the ON. After ischemic injury, the expression of Iba1 was dramatically enhanced in the ON, while the administration of OA significantly reduced the Iba1 level in the ON (Figure 10C,D). Furthermore, OA induced significantly more Ym1-positive cells (Figure 10A,B) and upregulated TGF-b expression in the ON after rAION induction (Figure 10C,E). These results indicate that OA-modulated microglia were polarized toward the anti-inflammatory M2 phenotype after optic nerve ischemic injury.

## 4. Discussion

Our morphologic data from this study clearly show that OA treatment enhanced RGC survival and significantly reduced the recruitment of inflammatory cells into the optic nerve after infarction. Furthermore, OA not only prevented RGC apoptosis but also preserved RGC function as demonstrated by FVEP, confirming its beneficial effect on the ischemic injury of the optic nerve. In addition, we observed that OA treatment ameliorated neuroinflammation and selectively polarized microglia toward an anti-inflammatory M2 status, resulting in a neuroprotective effect.

Post-ischemic inflammatory responses were identified in the rAION model, including the release of proinflammatory cytokines, extrinsic macrophage infiltration, intrinsic microglial activation, and the breakdown of the blood–optic nerve barrier [14,15,18,20,48]. ON ischemia triggered early inflammatory cytokine release, followed by subsequent extrinsic or intrinsic inflammatory cell invasion and activation, leading to axon dysfunction and RGC loss. Interleukin 6 (IL-6) acts as both a proinflammatory and neuropoietic cytokine involved in the development of the nervous system and neuron differentiation or degeneration. The role of IL-6 in RGC damage or protection remains controversial. Several studies have shown that a reduction in proinflammatory cytokines, including IL-6, IL-1β, inducible nitric oxidase synthase (iNOS), and tumor necrosis factor-α (TNF-α), improves RGC survival after optic nerve injury [34,49,50,51,52]. In the present study, we found that increased IL-6 immunostaining was mainly located in the RGC layers after ischemic injury (Figure 7C,F), and OA treatment could reduce the release of IL-6, which was shown to be synthesized in damaged RGCs and to be an important marker for the disruption of axonal transport [53]. OA further attenuates rAION-induced extrinsic macrophage invasion (Figure 7A,D) and intrinsic microglia activation (Figure 7B,E). These data indicate that OA blocks early ischemia-induced inflammatory responses, leading to the alleviation of optic disc swelling and preservation of RNFL thickness (Figure 4 and Figure 5).

Axon demyelination and/or focal damage to the ON has been identified in clinical NAION patients and NAION animal models [20,54,55]. RGC degeneration resulting from axon demyelination may be an important contributor to severe visual impairment. Direct changes in axon myelination were demonstrated by the CNPase immunostaining of the ON (Figure 8). Consistent with previous studies, we observed a markedly reduced CNPase staining of the ON after ischemic injury, and OA treatment maintained the level of CNPase staining in the infarcted ON, which indicates that OA significantly reduces ON demyelination and stabilizes the myelin sheath.

Microglia/macrophage-mediated neuroinflammation has been considered to be an important contributing factor in the pathogenesis of ischemic optic neuropathy [18,19,20,48]. In response to different stimuli, microglia can switch to two different phenotypes: M1 and M2. The classically activated M1 microglia, serving as the first line of defense, recognize deleterious stimuli and produce proinflammatory cytokines such as IL-1β, IL-6, iNOS, cyclooxygenase-2 (COX2), TNF-α, several chemokines, reactive oxygen species (ROS), and other toxic molecules [56,57,58,59]. The persistent activation of M1 microglia induces chronic inflammation, accelerating neurodegenerative processes. The polarization of alternatively activated M2 microglia occurs in response to specific anti-inflammatory factors, such as IL-4, IL-10, IL-13, and TGF-β. The M2 microglia-mediated induction of IL-4, Arg1, Ym1, and TGF-β suppresses inflammation and supports neuron differentiation, the repair or regeneration of the nervous system, and the restoration of tissue homeostasis [60,61,62,63]. In this study, we found that OA treatment not only diminished ischemia-induced ED1 and Iba1 expression but further induced the expression of M2 microglia markers, Ym1 and TGF-b, in the ON after infarct injury (Figure 10). These data suggest that OA can promote microglia/macrophage polarization into M2 microglia, suppressing inflammation and facilitating recovery following ON infarction.

Oxidative stress resulting from mitochondrial dysfunction, the overproduction of ROS, and an impaired antioxidant system contributes to the pathogenesis of many ocular diseases [64,65,66]. The nuclear factor erythroid 2-related factor (Nrf2) signaling pathway activated by oxidative stress regulates downstream antioxidant or detoxifying enzymes, resulting in anti-inflammatory and anti-apoptotic effects [24,67,68,69,70,71]. Our previous report demonstrated that the activation of the Nrf2 signaling pathway protects RGCs against ischemic injury through its anti-inflammatory and anti-apoptotic actions [14]. Moreover, recent reports indicated that the activation of Nrf2 signaling could inhibit M1-microglia-induced proinflammatory responses and polarize microglia/macrophages toward anti-inflammatory M2 microglia [72,73,74,75,76]. Our present data show that the Nrf2 pathway was significantly activated by OA treatment after rAION induction. These findings support the assertion that the beneficial effects of OA for ischemic optic neuropathy are associated with the activation of Nrf2 signaling, promoting M2 microglia polarization and attenuating the ischemia-induced inflammation and demyelination and apoptotic death of RGCs.

Base on the evidence from this study and our previous study [14], the data suggest activation of the Nrf2 signaling pathway promotes RGC survival and preserved the visual function after optic nerve ischemic injury. We will further evaluate effects of the Nrf2 activators, which have been used in the clinical trials for other indications, in this experimental ischemic optic neuropathy model to facilitate the translational application to NAION patients in the soon future.

## 5. Conclusions

**Figure 11:**
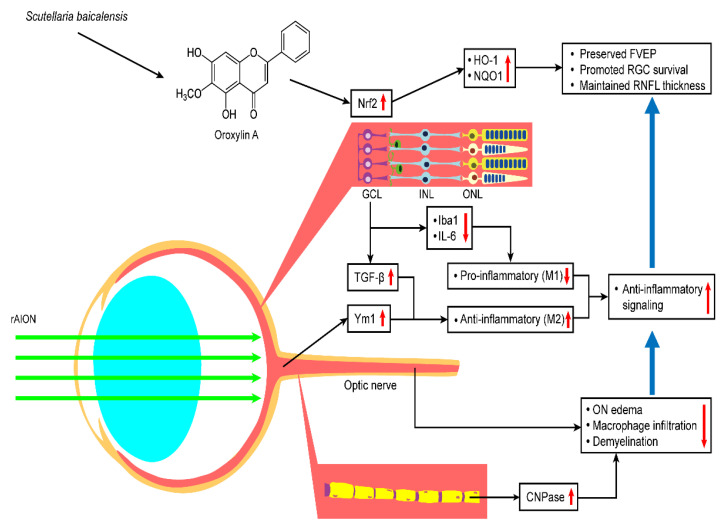
Hypothetical model of the protective effects of OA in the rAION model. OA treatment after rAION induction can enhance Nrf2 pathway activation and thereby preserve FVEP, promote RGC survival, and maintain RNFL thickness. OA also shows anti-inflammatory effects and modulates microglia polarization by increasing TGF-b and Ym-1 expression and decreasing Iba1 and IL-6 expression. OA protects the optic nerve by reducing ON edema, macrophage infiltration, and demyelination.

In summary, this study provides evidence that OA promotes retinal ganglion cell survival and preserves visual function by preventing the apoptosis of RGCs, maintaining myelin sheath integrity, reducing the levels of proinflammatory cytokines, and modulating microglial polarization. These neuroprotective effects were achieved by the activation of Nrf2 signaling in the retina (Figure 11). Our data indicate that OA has the potential to be a future therapeutic agent for ischemic optic neuropathy.

## Data Availability

All data generated or analyzed during this study are included in this publication article.
